# Comparative insights into *Fusobacterium nucleatum* and *Helicobacter pylori* in human cancers

**DOI:** 10.3389/fmicb.2025.1677795

**Published:** 2025-10-21

**Authors:** Alua Gusmaulemova, Botakoz Kurentay, Dina Bayanbek, Gulmira Kulmambetova

**Affiliations:** Department of Genomics, National Center for Biotechnology, Astana, Kazakhstan

**Keywords:** *Fusobacterium nucleatum*, *Helicobacter pylori*, colorectal cancer, gastric cancer, red meat, processed meat, virulence factors

## Abstract

*Fusobacterium nucleatum* and *Helicobacter pylori* are two microbial species increasingly recognized for their roles in gastrointestinal (GI) carcinogenesis, particularly in colorectal cancer (CRC) and gastric cancer (GC), respectively. While *H. pylori* has been long classified as a Group 1 carcinogen due to its well-characterized pathogenic mechanisms, *F. nucleatum* has more recently emerged as a key microbial contributor to CRC, with growing evidence linking it to tumor progression, immune evasion, and poor clinical outcomes. Despite occupying anatomically distinct niches within the GI tract, both bacteria converge on similar oncogenic pathways, including the activation of NF-κB signaling, β-catenin pathway dysregulation, and epithelial barrier disruption. In parallel, dietary factors – particularly the consumption of red and processed meats – contribute additional oncogenic pressure via carcinogenic compounds such as heme iron, N-nitroso compounds, and polycyclic aromatic hydrocarbons. These dietary components not only damage host tissue but may also potentiate bacterial virulence and promote microbial persistence. This review provides a comparative analysis of the oncogenic strategies employed by *F. nucleatum* and *H. pylori*, with an emphasis on their interactions with diet-derived carcinogens and implications for therapeutic interventions targeting the microbiota–diet–host axis in GI cancers.

## 1 Introduction

Gastrointestinal (GI) cancers, notably colorectal cancer (CRC) and gastric cancer (GC), remain among the most prevalent and lethal malignancies globally, accounting for over 1.5 million deaths annually (Singh, 2024). While genetic susceptibility and environmental exposures such as smoking and obesity are established contributors to GI tumorigenesis, increasing attention has turned to the gut microbiome as a dynamic and potentially modifiable factor in cancer development (Shen et al., 2025).

Among the vast array of microbial species inhabiting the GI tract, *Fusobacterium nucleatum* and *Helicobacter pylori* have emerged as key pathogens with oncogenic potential. *H. pylori* has been extensively studied and is classified by the International Agency for Research on Cancer (IARC) as a Group 1 carcinogen, responsible for the majority of non-cardia gastric cancer cases worldwide (Moss, 2017; Wroblewski et al., 2010). In contrast, *F. nucleatum*, an oral anaerobe frequently detected in colorectal tumors, has gained attention only in recent years due to its association with advanced disease stages, immune evasion, and poor response to therapy (Galasso et al., 2025).

Despite residing in different anatomical compartments – *H. pylori* in the acidic environment of the stomach and *F. nucleatum* in the anaerobic colon – both bacteria employ strikingly similar pathogenic strategies. These include the induction of chronic inflammation, disruption of epithelial junctions, activation of proliferative signaling pathways, and modulation of host immune responses (Ito et al., 2020; Ou et al., 2022). At the molecular level, both species manipulate key signaling hubs such as NF-κB and β-catenin, which serve as convergence points for microbial virulence and host cell transformation (Moss, 2017; Neagu et al., 2025).

Beyond microbial mechanisms, dietary exposures play a pivotal role in shaping both cancer risk and microbial dynamics. High intake of red and processed meats, for example, is consistently associated with elevated CRC and GC risk in epidemiological studies (Di et al., 2023). This association is partially attributed to the formation of procarcinogenic compounds such as polycyclic aromatic hydrocarbons (PAHs), heterocyclic amines (HCAs), heme iron, and N-nitroso compounds (NOCs) during meat processing and digestion. Emerging evidence suggests that these dietary carcinogens can act synergistically with microbial factors–either by enhancing virulence gene expression, promoting reactive oxygen species (ROS) production, or creating a tissue microenvironment conducive to bacterial persistence (Malesza et al., 2022; Momal et al., 2025).

In preparing this review, a targeted literature search was conducted to explore the oncogenic mechanisms of *Fusobacterium nucleatum* and *Helicobacter pylori* in gastrointestinal carcinogenesis. Relevant peer-reviewed publications were identified using databases such as PubMed, Scopus, and Google Scholar, covering the period from 2000 to 2025. The search focused on studies containing key terms including *Fusobacterium nucleatum*, *Helicobacter pylori*, colorectal cancer, gastric cancer, CagA, FadA, VacA, NF-κB, immune evasion, red meat, processed meat, dietary carcinogens, reactive oxygen species, gut microbiota, and tumor microenvironment. Articles were included if they were written in English and provided molecular, clinical, or epidemiological insights into inflammation-driven tumorigenesis, immune modulation, or diet-microbiota interactions. Studies unrelated to gastrointestinal cancers, outdated reviews lacking mechanistic relevance, or non-English publications were excluded. A total of 61 high-quality studies were selected based on relevance, scientific rigor, and thematic alignment with the aims of this review.

In this review, we systematically compare the oncogenic mechanisms of *F. nucleatum* and *H. pylori*, with a focus on their interactions with diet-derived carcinogens. We examine how chronic inflammation, immune modulation, and barrier dysfunction collectively drive carcinogenesis, and how microbial–dietary synergy exacerbates these processes. By delineating both converging and diverging pathways of microbial carcinogenesis, we aim to identify potential targets for microbiota-based prevention and treatment strategies in GI cancers.

## 2 Oncogenic mechanisms of *F. nucleatum* in colorectal cancer

The role of *Fusobacterium nucleatum* in colorectal cancer (CRC) has become increasingly evident over the past decade. Initially identified as an oral commensal, *F. nucleatum* is now recognized as a recurrent component of the CRC microbiota, frequently enriched in tumor tissues compared to adjacent normal mucosa. Its presence correlates with advanced tumor stage, lymph node metastasis, microsatellite instability, and poor patient outcomes (Dadgar-Zankbar et al., 2024; Kulmambetova et al., 2023; McIlvanna et al., 2021). Mechanistically, *F. nucleatum* promotes tumorigenesis via three interconnected pathways: chronic inflammation, dysregulation of epithelial signaling, and suppression of antitumor immune responses. These effects are largely mediated by specific virulence factors, including lipopolysaccharide (LPS), FadA adhesin, and Fap2 protein (Rubinstein et al., 2013).

### 2.1 Chronic inflammation and TLR4/NF-kB signaling

A central mechanism by which *F. nucleatum* contributes to colorectal tumor development is through the induction of chronic inflammation. Its LPS component engages Toll-like receptor 4 (TLR4) on colonic epithelial cells and innate immune cells, triggering a MyD88-dependent signaling cascade that culminates in the activation of the NF-κB transcription factor (Yang et al., 2017). Once activated, NF-κB translocates to the nucleus and drives the expression of pro-inflammatory cytokines such as IL-6, IL-1β, TNF-α, and IL-17. These cytokines collectively foster a microenvironment conducive to tumor initiation and progression by promoting epithelial proliferation, angiogenesis, and immune cell recruitment (Wei et al., 2023).

**FIGURE 1:**
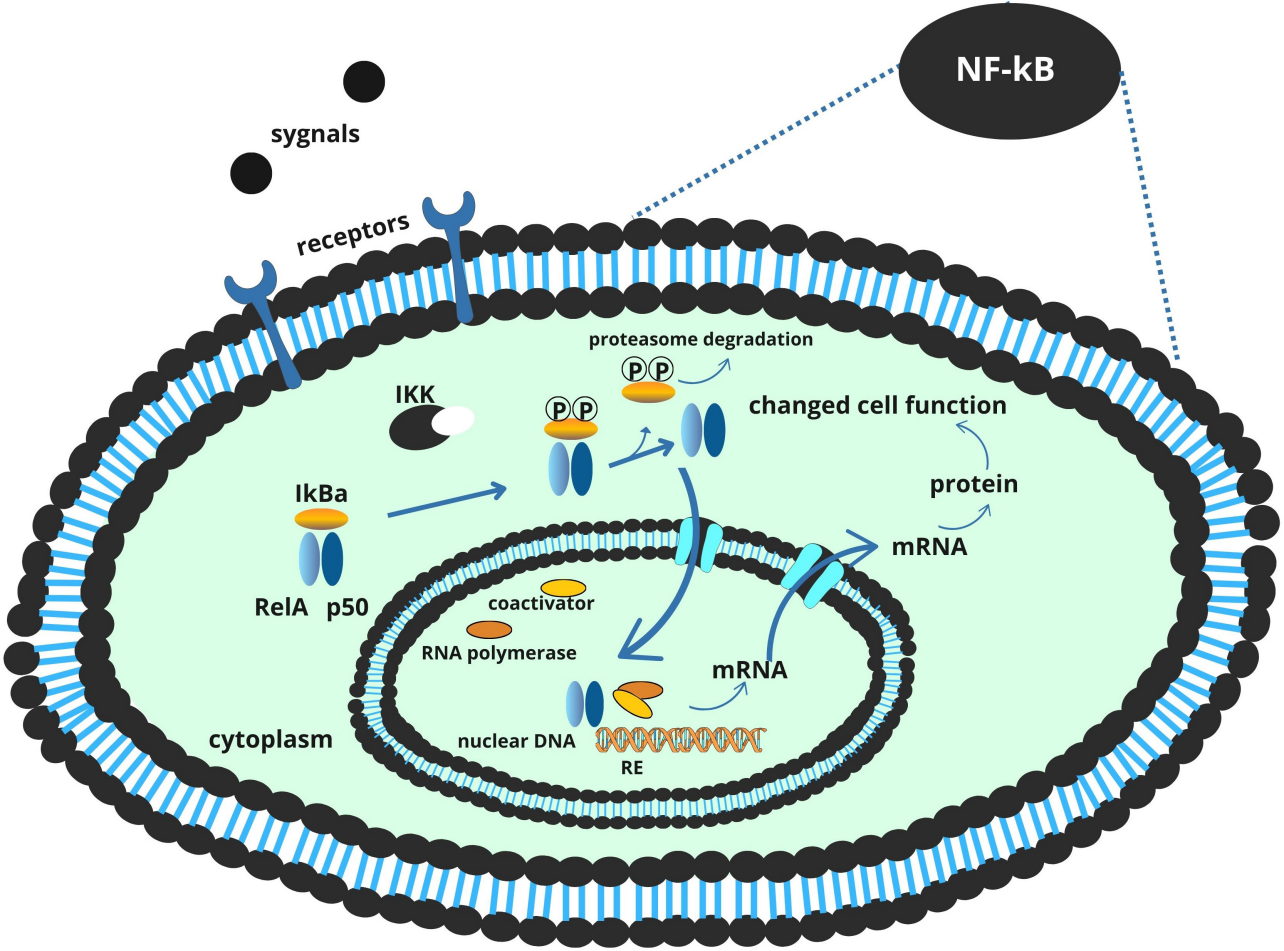
NF-κB signaling pathway and its role in inflammation and tumorigenesis.

This inflammatory signaling is further amplified by dietary factors. Heme iron, abundant in red meat, promotes the formation of reactive oxygen species (ROS), which not only cause direct DNA damage in colonocytes but also serve as secondary activators of NF-κB. The resulting oxidative stress reinforces the inflammatory loop initiated by *F. nucleatum*, establishing a chronic, tumor-promoting environment (Kang et al., 2019; Lee et al., 2023; Malesza et al., 2022; Figure 1).

### 2.2 Proliferation and β-catenin pathway disruption via FadA

**FIGURE 2:**
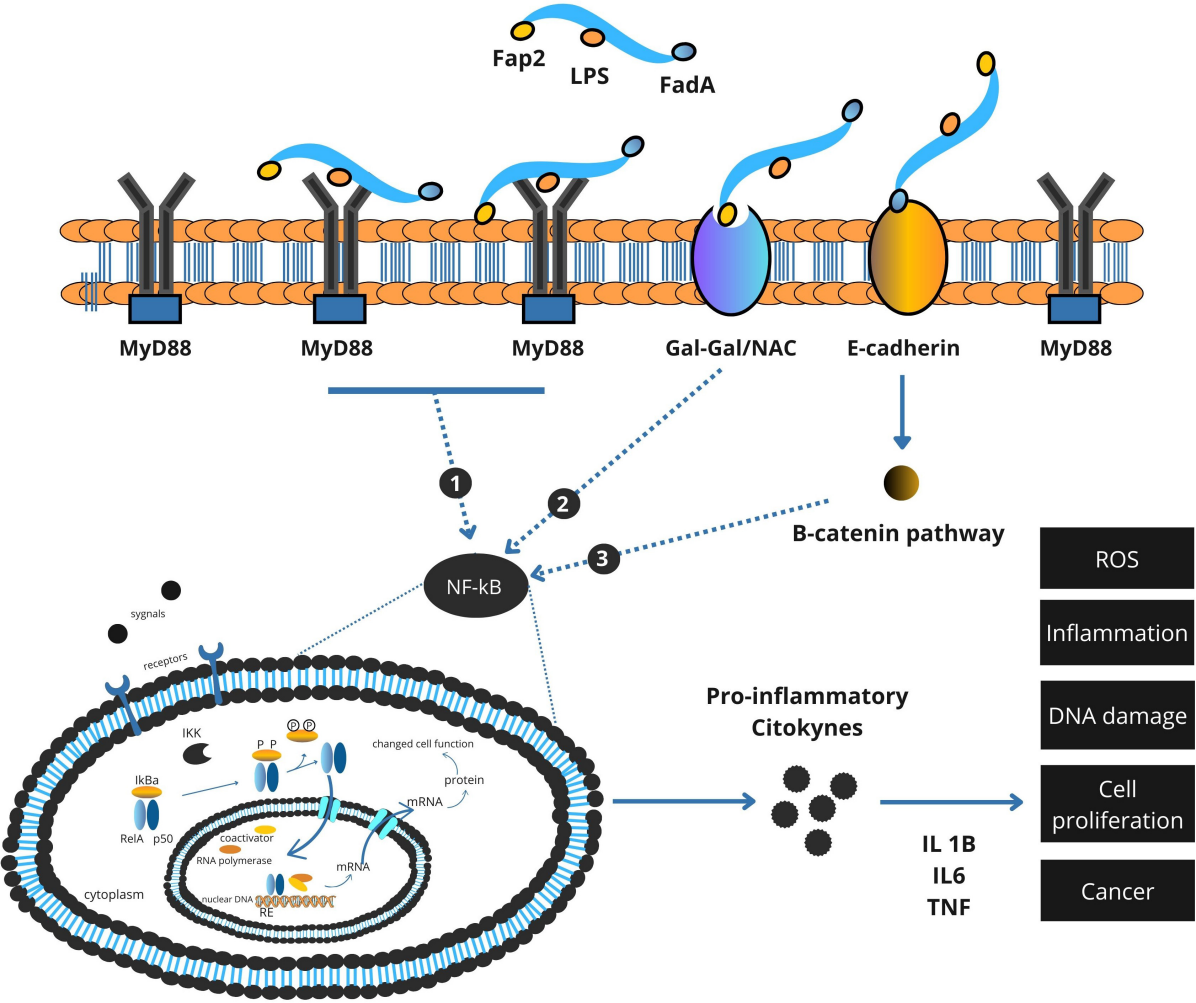
Integrated model of *Fusobacterium nucleatum* virulence factor–mediated activation of NF-κB and β-catenin signaling in colorectal cancer.

In addition to promoting inflammation, *F. nucleatum* directly perturbs epithelial cell signaling through the action of FadA, an adhesin expressed on its surface. FadA binds to E-cadherin on the surface of colonic epithelial cells, leading to the disassembly of the E-cadherin–β-catenin complex (Figure 2). This interaction results in the nuclear translocation of β-catenin, a transcriptional co-activator implicated in Wnt signaling. Within the nucleus, β-catenin induces the expression of genes associated with cell proliferation and survival, including *c-Myc* and *cyclin D1*. This aberrant activation of proliferative pathways facilitates uncontrolled cell division and contributes to adenoma-to-carcinoma progression (Fardini et al., 2011; Rubinstein et al., 2013).

Importantly, the epithelial-disruptive effects of FadA can be potentiated by dietary carcinogens. N-nitroso compounds (NOCs), formed during the digestion of processed meats or endogenously in the colon, have been shown to cause DNA alkylation and mutation of tumor suppressor genes. In combination with FadA-induced β-catenin signaling, these mutagenic insults may accelerate cellular transformation (Galasso et al., 2025).

### 2.3 Immune evasion via Fap2-TIGIT interaction

Effective antitumor immunity relies on the activity of cytotoxic lymphocytes, including natural killer (NK) cells and CD8+ T cells. *F. nucleatum* circumvents immune surveillance by expressing Fap2, a surface protein that binds to the T cell immunoreceptor with Ig and ITIM domains (TIGIT), an inhibitory receptor on NK and T cells. This interaction mimics immune checkpoint signaling, suppressing cytotoxic activity and allowing tumor cells to evade immune-mediated destruction (Coppenhagen-Glazer et al., 2015; Gur et al., 2015).

Furthermore, dietary patterns that favor *F. nucleatum* persistence may exacerbate this immune evasion. High consumption of processed meats is associated with a depletion of beneficial gut commensals such as *Bifidobacterium*, and a corresponding enrichment of pro-inflammatory or opportunistic microbes, creating a microbiota environment that supports *F. nucleatum* colonization and immune resistance (Wang Y. et al., 2023).

### 2.4 Integrated model of *F. nucleatum* –mediated carcinogenesis

Taken together, the oncogenic potential of *F. nucleatum* in CRC is mediated by a coordinated network of molecular interactions. Through LPS-TLR4 signaling, it establishes a chronic inflammatory state. Via FadA, it disrupts epithelial signaling and promotes hyperproliferation. And through Fap2, it silences antitumor immune responses (Coppenhagen-Glazer et al., 2015; Rubinstein et al., 2013). These effects are further modulated by host dietary exposures, particularly red and processed meat intake, which not only provide pro-carcinogenic compounds but may also enhance bacterial virulence and survival (Ungvari et al., 2025).

**FIGURE 3:**
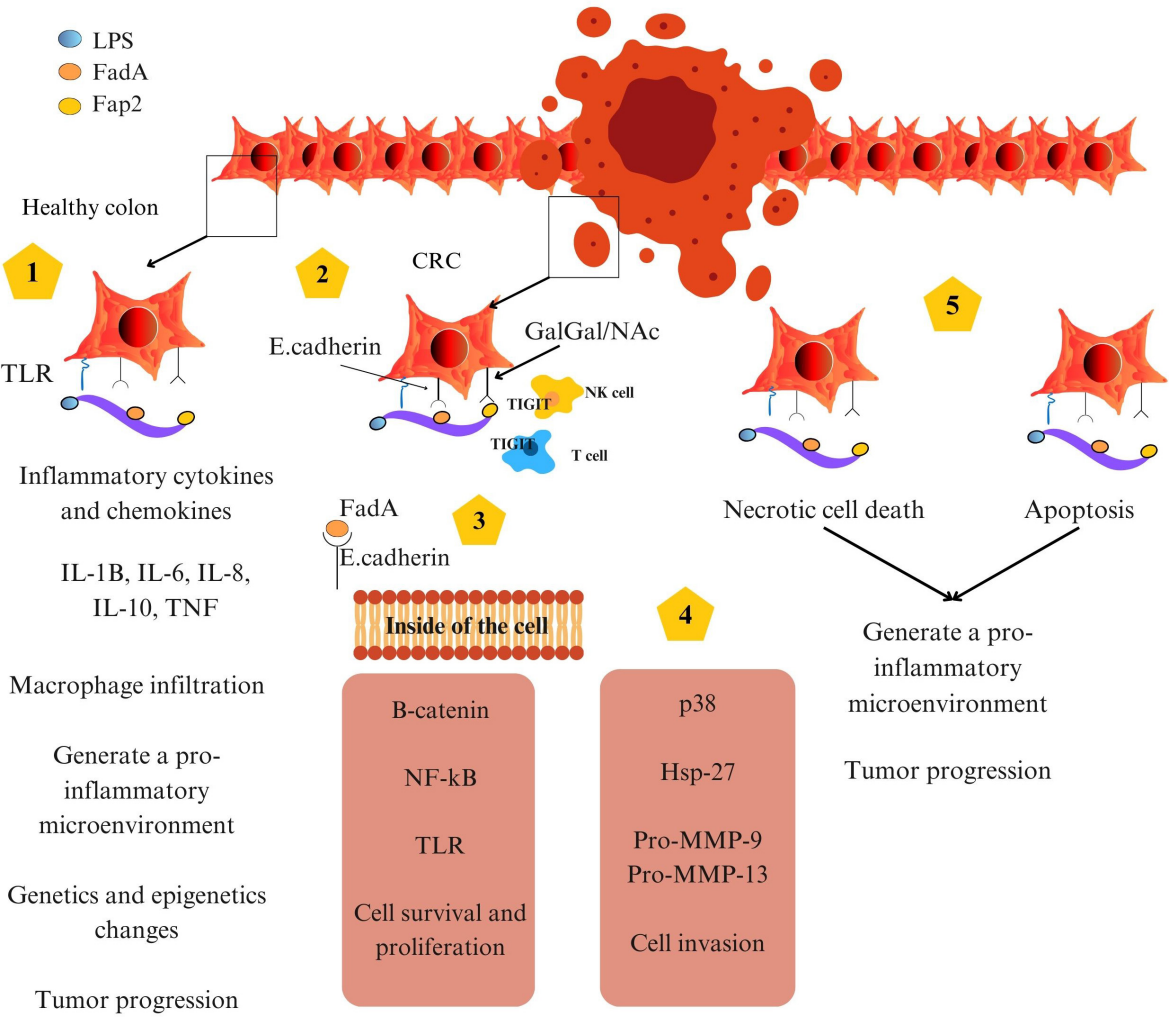
Schematic representation of CagA-induced signaling pathway in gastric epithelial cells.

A comprehensive summary of these oncogenic mechanisms is provided in Figure 3, which integrates *F. nucleatum*’s roles in inflammation, epithelial disruption, immune evasion, and cell death within the tumor microenvironment.

## 3 Oncogenic mechanisms of *Helicobacter pylori* in gastric cancer

*Helicobacter pylori* is a Gram-negative, spiral-shaped bacterium that colonizes the gastric mucosa of nearly half the global population (Wroblewski et al., 2010). Its persistent infection is the primary etiological factor in non-cardia gastric cancer and has earned its classification as a Group 1 carcinogen by the IARC (Ono et al., 2025). The oncogenic capacity of *H. pylori* arises from its unique ability to colonize the harsh gastric environment, disrupt epithelial integrity, hijack proliferative signaling pathways, and evade immune surveillance. These effects are mediated by a suite of well-characterized virulence factors, including urease, cytotoxin-associated gene A (CagA), and vacuolating cytotoxin A (VacA) (Alipour, 2021; Cooke et al., 2013).

### 3.1 Colonization, mucosal damage and role of urease

The survival of *H. pylori* in the highly acidic environment of the stomach depends on its production of urease, an enzyme that hydrolyzes urea into ammonia and carbon dioxide. This local neutralization of gastric acid creates a hospitable niche within the mucus layer of the gastric epithelium, facilitating bacterial colonization. However, the process of colonization itself contributes to mucosal injury. Ammonia, a byproduct of urease activity, is cytotoxic at high concentrations and disrupts tight junction integrity, enhancing paracellular permeability. This damage promotes further bacterial adherence and translocation of virulence factors into epithelial cells (Ansari and Yamaoka, 2017).

Notably, high dietary salt intake exacerbates this mucosal vulnerability. Hyperosmolar conditions induced by excess salt have been shown to upregulate the expression of *H. pylori* virulence genes, including *cagA* and *vacA*, while simultaneously weakening mucosal defenses. Salt-induced epithelial damage thus acts as both an enabler and amplifier of bacterial pathogenesis, particularly in the context of diets rich in processed meats (Ansari and Yamaoka, 2017; Jones et al., 2010; Kim et al., 2019).

### 3.2 CagA-dependent pathogenesis: manipulation of proliferative pathways

The CagA oncoprotein is the most extensively studied virulence factor of *H. pylori* (Kim et al., 2015). Delivered directly into gastric epithelial cells via a type IV secretion system (T4SS), CagA exerts multifaceted effects on host cell behavior depending on its phosphorylation status (Wang H. et al., 2023).

**FIGURE 4:**
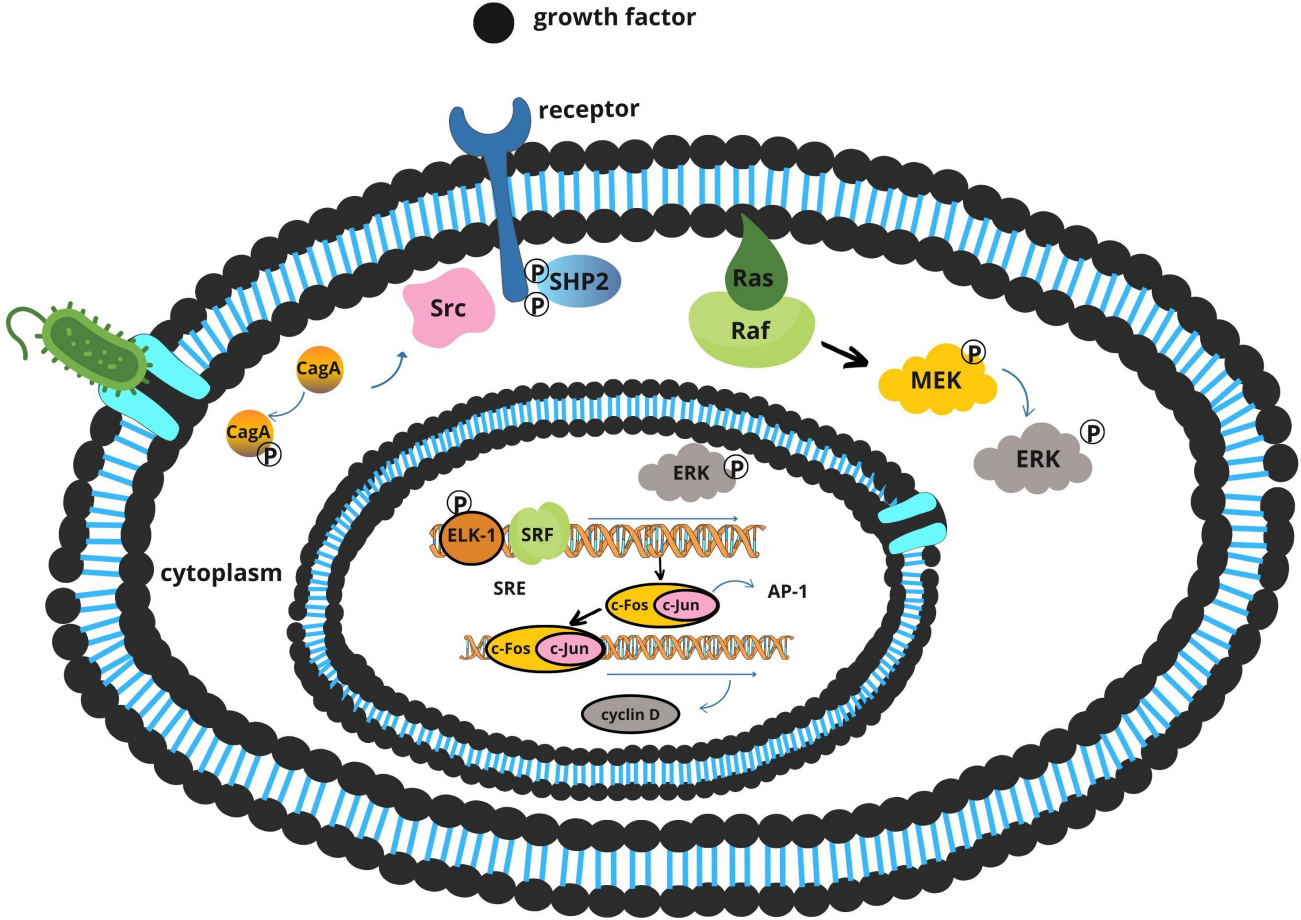
Proposed mechanisms of *Fusobacterium nucleatum* in colorectal carcinogenesis.

Once translocated, CagA is phosphorylated by host Src and Abl tyrosine kinases. The phosphorylated form interacts with SHP-2, a protein tyrosine phosphatase that activates the Ras–ERK signaling cascade (Figure 4). This results in increased cell proliferation, morphological transformation, and disruption of cellular polarity (Alipour, 2021; Tsutsumi et al., 2003).

**FIGURE 5:**
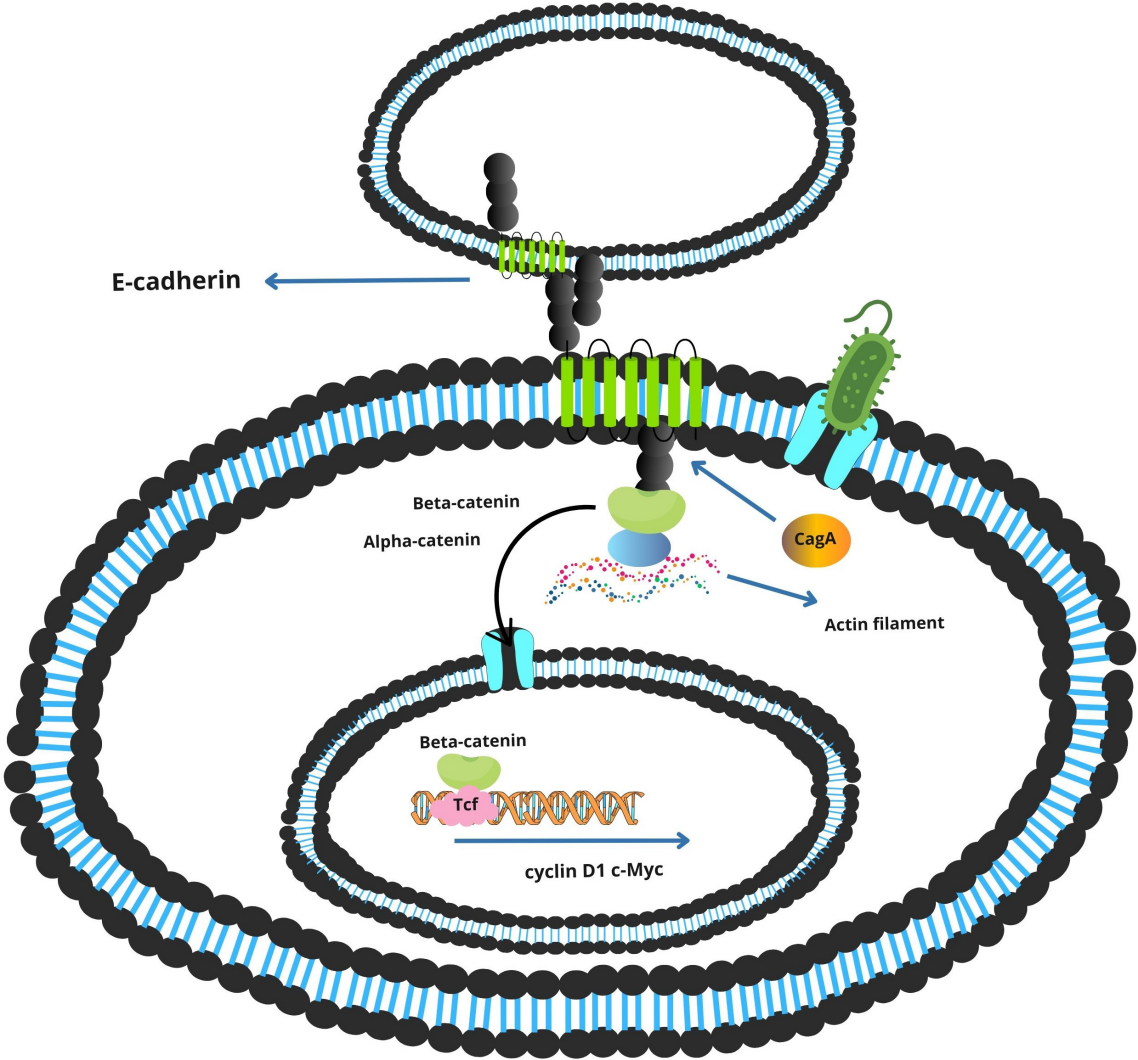
Activation of the E-cadherin/β-catenin signaling pathway by *Helicobacter pylori* CagA.

Simultaneously, the non-phosphorylated form of CagA perturbs adherens junctions by binding to E-cadherin, destabilizing the β-catenin complex (Figure 5). Released β-catenin translocates into the nucleus, where it activates Wnt target genes such as *cyclin D1* and *c-Myc*, promoting uncontrolled cellular proliferation (Jones et al., 2010; Wang H. et al., 2023).

The tumorigenic potential of CagA is further enhanced by dietary carcinogens. Polycyclic aromatic hydrocarbons (PAHs), commonly formed during high-temperature cooking of meats, can independently activate the ERK pathway and synergize with CagA-mediated signaling, intensifying proliferative and anti-apoptotic responses (Balendra et al., 2023; Jones et al., 2010; Twarda-Clapa et al., 2022).

### 3.3 VacA-mediated immune modulation and persistence

VacA, a pore-forming exotoxin secreted by *H. pylori*, contributes to both immune evasion and tissue damage. Once internalized, VacA forms anion-selective channels in host membranes, leading to cellular vacuolization and mitochondrial dysfunction (Jones et al., 2010).

In immune cells, VacA inhibits T-cell activation by interfering with the calcineurin–NFAT signaling axis, reducing IL-2 production and impairing cytotoxic responses. It also induces apoptosis in T lymphocytes and other immune cell subsets, contributing to long-term immune suppression and bacterial persistence (Boncristiano et al., 2003; Gebert et al., 2003).

Interestingly, the genotoxic effects of dietary nitrosamines–common in processed meats–appear to parallel and enhance VacA activity. These compounds selectively damage normal gastric epithelial cells while allowing pre-malignant cells to evade apoptosis, fostering clonal expansion in a dysregulated environment (Szabò et al., 1999; Zhao et al., 2017).

### 3.4 Integrated model of *H. pylori*–induced gastric carcinogenesis

The pathogenesis of *H. pylori*-associated gastric cancer involves a stepwise progression from chronic gastritis to atrophic gastritis, intestinal metaplasia, dysplasia, and ultimately adenocarcinoma. This cascade is driven by a persistent cycle of mucosal damage, immune modulation, and aberrant proliferation (Cooke et al., 2013; Moss, 2017).

Urease enables colonization, CagA activates proliferative and survival pathways, and VacA disrupts immune surveillance and epithelial integrity. When these bacterial effects are compounded by dietary factors – particularly salt, PAHs, and nitrosamines – the risk of malignant transformation increases substantially (Diggs et al., 2011; Zhu et al., 2013).

Figure 5 presents a schematic overview of these mechanisms, highlighting the interplay between *H. pylori* and virulence factors in the gastric tumor microenvironment.

## 4 Comparative analysis: converging and diverging oncogenic strategies

Despite inhabiting distinct regions of the gastrointestinal (GI) tract and employing different mechanisms of host interaction, *Fusobacterium nucleatum* and *Helicobacter pylori* exhibit remarkable convergence in their capacity to drive tumorigenesis. Both bacteria exploit the host’s immune and signaling pathways to promote chronic inflammation, disrupt epithelial homeostasis, and enhance cellular proliferation. Understanding these converging and diverging mechanisms is critical for identifying shared therapeutic targets and pathogen-specific intervention strategies.

### 4.1 Converging mechanisms: common pathways to oncogenesis

Both *F. nucleatum* and *H. pylori* co-opt host signaling pathways that are central to inflammation and tumor promotion (Table 1). Specifically, they converge on two key molecular axes: the NF-κB pathway and the Wnt/β-catenin signaling cascade (Galasso et al., 2025; Ito et al., 2020).

**TABLE 1:** Summary of bacterial virulence factors and interleukin interactions in CRC and GC.

Bacterium	Affected interleukins	Virulence factor	Functional effect in CRC/GC	Expression pattern	References
*F. nucleatum*	IL-6, IL-8	FadA	Activates E-cadherin/β-catenin pathway, induces IL-8 release, promotes inflammation and proliferation (CRC)	↑	Ansari and Yamaoka, 2017; Lee et al., 2023
*F. nucleatum*	IL-10, IL-6	Fap2	Immune evasion via TIGIT binding, modulates IL-10 to suppress immune response, contributes to CRC progression	↑/↓	Gur et al., 2015; Momal et al., 2025
*H. pylori*	IL-8, IL-6	CagA	Induces IL-8 via NF-κB, promotes angiogenesis and proliferation in GC, alters microenvironment	↑	Cooke et al., 2013; Farvid et al., 2021
*H. pylori*	IL-10, IL-1β	VacA	Suppresses immune response (↑IL-10), induces IL-1β, promotes apoptosis and immune tolerance in GC	↑/↓	Cooke et al., 2013; Ito et al., 2020; Wang Y. et al., 2023

Arrows indicate the direction of interleukin expression change: ↑ denotes upregulation (increased expression/activity), ↓ denotes downregulation (decreased expression/activity).

In *F. nucleatum*, activation of NF-κB is initiated by lipopolysaccharide (LPS) recognition via TLR4 on epithelial and immune cells, resulting in the transcription of pro-inflammatory cytokines (e.g., IL-6, TNF-α) (Kang et al., 2019). *H. pylori*, although lacking classical LPS-TLR4 stimulation, activates NF-κB through intracellular pattern recognition receptors such as NOD1, which senses bacterial peptidoglycan following CagA-mediated cell manipulation. In both cases, chronic NF-κB signaling creates a pro-tumorigenic inflammatory microenvironment characterized by immune cell infiltration, angiogenesis, and oxidative stress (Wang H. et al., 2023).

Both pathogens disrupt E-cadherin-mediated cell-cell adhesion to activate β-catenin-dependent transcription. *F. nucleatum* achieves this through its FadA adhesin binding to E-cadherin, while *H. pylori* employs the non-phosphorylated form of CagA to interfere with the same complex. The downstream consequences are similar: nuclear accumulation of β-catenin, upregulation of *c-Myc* and *cyclin D1*, and enhanced epithelial proliferation (Fardini et al., 2011; Tsutsumi et al., 2003).

A critical point of convergence is the interaction with dietary carcinogens. Both bacteria operate within microenvironments shaped by red and processed meat consumption. Dietary heme iron, PAHs, and nitrosamines potentiate ROS production, which in turn amplifies NF-κB and Wnt pathway signaling. This synergy escalates DNA damage, promotes epithelial turnover, and facilitates clonal selection of transformed cells (Ferro et al., 2020; Lee et al., 2023; Oostindjer et al., 2014).

These overlapping mechanisms suggest the existence of shared “oncogenic chokepoints” in the GI mucosa – molecular nodes that are repeatedly exploited by different pathogens and dietary carcinogens to drive malignancy.

### 4.2 Diverging strategies: niche adaptation and toxin delivery

While *F. nucleatum* and *H. pylori* share several functional outcomes, their strategies of achieving these effects are fundamentally distinct, reflecting their adaptation to specific anatomical and microbial contexts.

*Helicobacter pylori* uses a type IV secretion system (T4SS) to inject the CagA protein directly into gastric epithelial cells. This allows for precise manipulation of host signaling in a contact-dependent and cell-specific manner (Tsutsumi et al., 2003). In contrast, *F. nucleatum* exerts its effects extracellularly, relying on surface adhesins like FadA and Fap2 to bind host receptors and modulate signaling cascades indirectly (Dadgar-Zankbar et al., 2024). This difference in delivery likely contributes to the more chronic and opportunistic nature of *F. nucleatum*-mediated pathogenesis.

*Fusobacterium nucleatum* subverts antitumor immunity primarily through immune checkpoint mimicry. Its Fap2 protein binds TIGIT, an inhibitory receptor on NK and T cells, suppressing cytotoxic activity and enabling immune evasion (Gur et al., 2015). *H. pylori*, on the other hand, employs the VacA toxin to directly impair T-cell activation by targeting intracellular calcium signaling and inducing apoptosis (Boncristiano et al., 2003). These divergent strategies result in distinct patterns of immune tolerance and inflammation within their respective tissue microenvironments.

## 5 Synergistic role of dietary and environmental factors

Diet plays a critical role in shaping the risk landscape for gastrointestinal (GI) cancers, both by directly influencing host physiology and by modulating the composition and behavior of the gut microbiota (Bouvard et al., 2015). Among dietary exposures, the consumption of red and processed meats is consistently linked to increased incidence of colorectal cancer (CRC) and, to a lesser extent, gastric cancer (GC). These associations are supported by robust epidemiological data and underpinned by well-characterized molecular mechanisms involving diet-derived carcinogens, host inflammation, and microbial synergy (Alisson-Silva et al., 2016; Benarba, 2018; Cross et al., 2007).

### 5.1 Epidemiological evidence linking meat consumption to GI cancers

#### 5.1.1 Data on colorectal cancer

Numerous large-scale epidemiological studies and meta-analyses consistently associate the consumption of red and processed meats with an increased risk of CRC (Larsson and Wolk, 2006). Evidence from major cohort studies, like the Million Women Study involving over 540,000 participants, first highlighted this significant association (Papier et al., 2025). These findings have been powerfully reinforced by comprehensive meta-analyses (Ungvari et al., 2025). For instance, a review of 148 prospective studies confirmed elevated risks for colorectal (RR = 1.17), colon (RR = 1.21), and rectal cancers (Farvid et al., 2021), a conclusion mirrored in another analysis of nearly 4 million people that underscored the consistency of this link across global populations (Di et al., 2023; Kulmambetova et al., 2024; Ono et al., 2025).

Furthermore, the risk appears to be dose-dependent, with research establishing that consuming as little as 100 g/day of red meat or 50 g/day of processed meat leads to a measurable increase in CRC risk (Chan et al., 2011). Collectively, these extensive data provide the scientific basis for the IARC’s classification of processed meat as a definite (Group 1) and red meat as a probable (Group 2A) carcinogen (Moss, 2017).

#### 5.1.2 Data on gastric cancer

Although epidemiological evidence is more robust for colorectal cancer, multiple studies also implicate red and processed meat consumption as risk factors for gastric cancer (GC). A meta-analysis by Zhu et al. (2013) reported that high intake of red or processed meat was associated with a 45% increased risk of gastric cancer. Dietary analyses from a broader cohort corroborate these findings, indicating that processed meat intake is positively associated with non-cardia GC, though associations with red meat alone may be weaker or inconsistent (Ferro et al., 2020).

In aggregate, Kim et al. (2019) conducted a meta-analysis including 5 cohorts and 19 case–control studies (9,726 cases), revealing that subjects with high red meat intake had a 41% higher risk of gastric cancer, while high processed meat intake conferred a 57% increased risk. Notably, dose–response analysis showed a 26% increased GC risk per 100 g/day increment in red meat consumption (Kim et al., 2010, 2019).

These associations appear particularly strong in case–control studies, whereas many cohort investigations yield null or weaker findings, especially for gastric cardia and non-cardia subtypes, highlighting heterogeneity across study designs and geographic regions.

### 5.2 Molecular mechanisms of dietary carcinogens

Mechanistically, the carcinogenic potential of red and processed meats is driven by several bioactive compounds formed during cooking and processing. High-temperature cooking methods generate heterocyclic amines (HCAs) and polycyclic aromatic hydrocarbons (PAHs) – both of which are metabolically activated into DNA-damaging agents. HCAs such as MeIQx and PhIP are hydroxylated by cytochrome P450 enzymes into reactive intermediates that form DNA adducts, particularly in colon epithelial cells, leading to mutagenesis (Cascella et al., 2018; Twarda-Clapa et al., 2022). PAHs like benzo[a]pyrene follow similar metabolic routes, producing genotoxic metabolites that interact with DNA and upregulate carcinogenic signaling pathways such as NF-κB and aryl hydrocarbon receptor signaling (Diggs et al., 2011; Huderson et al., 2013).

Heme iron, abundant in red meat, further exacerbates this risk. Its redox activity promotes the formation of reactive oxygen species (ROS), which trigger lipid peroxidation and produce mutagenic aldehydes such as malondialdehyde (MDA) and 4-hydroxynonenal (4-HNE), both implicated in colonocyte damage and tumor initiation (Szabò et al., 1999). In addition, heme iron facilitates the endogenous formation of N-nitroso compounds (NOCs), which alkylate DNA bases and have been shown to induce KRAS mutations, a hallmark of CRC (Cascella et al., 2018; Malesza et al., 2022). Nitrite and nitrate preservatives in processed meats also contribute to NOC formation, particularly under acidic gastric conditions, enhancing genotoxicity (Crowe et al., 2019).

A more recently discussed factor is N-glycolylneuraminic acid (Neu5Gc), a non-human sialic acid prevalent in red meat. Humans can incorporate Neu5Gc into their cell membranes, where it is recognized as foreign by circulating anti-Neu5Gc antibodies. This immune recognition may trigger chronic inflammation (xenosialitis), a proposed mechanism linking Neu5Gc accumulation to tumor progression in the colon (Cascella et al., 2018). Notably, poultry and fish-lacking Neu5Gc do not show the same carcinogenic profile, further strengthening this dietary specificity (Alisson-Silva et al., 2016).

### 5.3 Mechanistic synergy: How dietary factors potentiate bacterial virulence?

One of the most compelling emerging concepts in GI oncology is the functional synergy between dietary carcinogens and microbial pathogens. Both *F. nucleatum* and *H. pylori* rely on host inflammation, oxidative stress, and disrupted epithelial signaling to promote carcinogenesis conditions that are exacerbated by diet (Galasso et al., 2025; Ito et al., 2020).

Red meat–derived heme iron enhances ROS production in the gut, reinforcing NF-κB activation initiated by bacterial LPS (in *F. nucleatum*) or NOD1 signaling (in *H. pylori*). The resulting oxidative stress contributes to DNA damage and creates a pro-inflammatory niche favorable to bacterial persistence (Macho-González et al., 2020).

N-nitroso compounds and PAHs potentiate Wnt/β-catenin signaling, mirroring the effects of FadA (from *F. nucleatum*) and CagA (from *H. pylori*) on epithelial cells. This convergence amplifies the expression of oncogenes and anti-apoptotic factors, accelerating the progression from dysplasia to carcinoma (Huderson et al., 2013; Lee et al., 2023).

Processed meats alter the gut microbiome by depleting protective species such as *Bifidobacterium* and expanding pro-inflammatory taxa. These changes may promote colonization or overgrowth of *F. nucleatum*, which thrives in low-diversity, inflammation-prone environments (Lee et al., 2023; Ma et al., 2017).

### 5.4 The impact of other lifestyle factors: obesity, smoking, and alcohol

In addition to bacterial virulence and dietary carcinogens, obesity, smoking, and alcohol consumption are significant risk factors for gastrointestinal cancers. A Mendelian randomization study demonstrated a causal association between increased waist-to-hip ratio and colorectal cancer (OR ≈ 1.38), as well as a suggestive link to smoking (Li et al., 2024). A meta-analysis found that alcohol consumption elevated gastric cancer risk by approximately 39 % (OR = 1.39) (Ma et al., 2017). Moreover, obesity in combination with smoking or heavy alcohol use further increases gastric cancer susceptibility by up to 19 % (Lim et al., 2022). At a population level, smoking accounts for 43 % of GI cancer mortality, alcohol for 21 %, and elevated BMI for approximately 20 % (Danpanichkul et al., 2025). These host and lifestyle factors likely interact with microbial and dietary mechanisms to exacerbate oncogenesis and should be considered in multifactorial prevention models.

## 6 Discussion

This review has examined the distinct yet converging roles of *Fusobacterium nucleatum* and *Helicobacter pylori* in gastrointestinal (GI) carcinogenesis, highlighting their shared molecular targets, niche-specific adaptations, and synergistic interactions with dietary carcinogens. A central theme emerging from this comparative analysis is the functional redundancy by which unrelated bacterial species exploit common vulnerabilities in the host to promote malignant transformation.

Despite significant differences in ecological localization and virulence delivery, both pathogens converge on two key signaling hubs: NF-κB and β-catenin/Wnt. These pathways not only mediate inflammatory and proliferative responses but also serve as integration points for dietary carcinogens such as heme iron, polycyclic aromatic hydrocarbons (PAHs), and N-nitroso compounds (NOCs) (Kang et al., 2019; Rubinstein et al., 2013). The resulting molecular crosstalk creates a self-reinforcing loop of microbial virulence, host tissue damage, and environmental exposure – an axis that significantly accelerates tumor initiation and progression.

Importantly, the synergy between bacterial factors and diet is not merely additive but mechanistically intertwined. For example, FadA- or CagA-mediated disruption of epithelial integrity increases cellular vulnerability to ROS and DNA alkylation induced by red meat–derived compounds. Conversely, dietary elements can enhance microbial virulence: salt exposure upregulates *H. pylori*’s expression of CagA and VacA, while iron-rich diets promote *F. nucleatum* persistence through microbiota remodeling and oxidative stress. These findings support a “triad model” of carcinogenesis, wherein pathogen, host, and diet form an interactive network rather than operating as independent risk factors (Alisson-Silva et al., 2016; Balendra et al., 2023; Cooke et al., 2013; Dadgar-Zankbar et al., 2024).

From a clinical perspective, these insights present both challenges and opportunities. The overlapping pathways exploited by *F. nucleatum* and *H. pylori* underscore the potential of shared therapeutic targets, such as inhibitors of NF-κB or modulators of β-catenin activity. Such interventions could, in theory, be effective across multiple cancer types and microbial contexts. Second, the divergence in delivery systems and immune evasion tactics suggests that pathogen-specific strategies – e.g., T4SS blockers for *H. pylori*, TIGIT inhibitors for *F. nucleatum* – may enhance therapeutic precision and reduce off-target effects (Gur et al., 2015; Larsson and Wolk, 2006). Third, the strong interaction between dietary carcinogens and microbial virulence highlights the importance of integrated prevention models. Modifying meat intake, especially processed and high-heat-cooked products, may not only reduce direct carcinogen exposure but also attenuate microbial-driven pathogenesis (Crowe et al., 2019; Lee et al., 2023). Finally, the role of the microbiome as a dynamic and modifiable risk factor suggests avenues for prebiotic, probiotic, or vaccine-based interventions. For example, reducing colonization by virulent *F. nucleatum* strains or altering microbial community structures that support *H. pylori* persistence could represent adjunctive strategies in high-risk populations.

Nevertheless, several key research gaps remain. Most studies evaluating microbial–diet interactions are either cross-sectional or based on animal models, limiting causal inference. Future work should prioritize longitudinal cohort studies incorporating microbiome sequencing, dietary profiling, and host genomics; functional analyses of virulence gene expression in response to dietary components; and evaluation of interventional strategies that simultaneously target microbial and dietary drivers of carcinogenesis.

Moreover, the broader context of microbial consortia and community-level interactions – beyond *F. nucleatum* and *H. pylori* – remains underexplored. GI cancers likely result from the cumulative effects of microbial networks, not single pathogens, and future models should account for these complex ecological dynamics.

## 7 Conclusion

Comparative insights into the oncogenic mechanisms of *Fusobacterium nucleatum* and *Helicobacter pylori* reveal a shared ability to integrate microbial virulence with dietary and host-derived factors, amplifying gastrointestinal cancer risk. Recognition of these parallels underscores the importance of microbiome-targeted diagnostics and therapeutics. By leveraging existing strategies developed for *H. pylori*, novel interventions against *F. nucleatum*-associated colorectal cancer may be developed, offering promising avenues for integrated cancer prevention approaches.
